# Single Cell Analysis Linking Ribosomal (r)DNA and rRNA Copy Numbers to Cell Size and Growth Rate Provides Insights into Molecular Protistan Ecology

**DOI:** 10.1111/jeu.12425

**Published:** 2017-06-09

**Authors:** Rao Fu, Jun Gong

**Affiliations:** ^1^ Yantai Institute of Coastal Zone Research Chinese Academy of Sciences Yantai 264003 China; ^2^ University of Chinese Academy of Sciences Beijing 100049 China; ^3^ Laboratory of Microbial Ecology and Matter Cycles School of Marine Sciences Sun Yat‐Sen University Zhuhai 519082 China

**Keywords:** Activity, body, Body size, copy number variation, experimental warming, nutrient cycling, ribosome content

## Abstract

Ribosomal (r)RNA and rDNA have been golden molecular markers in microbial ecology. However, it remains poorly understood how ribotype copy number (CN)‐based characteristics are linked with diversity, abundance, and activity of protist populations and communities observed at organismal levels. Here, we applied a single‐cell approach to quantify ribotype CNs in two ciliate species reared at different temperatures. We found that in actively growing cells, the per‐cell rDNA and rRNA CNs scaled with cell volume (CV) to 0.44 and 0.58 powers, respectively. The modeled rDNA and rRNA concentrations thus appear to be much higher in smaller than in larger cells. The observed rRNA:rDNA ratio scaled with CV ^0.14^. The maximum growth rate could be well predicted by a combination of per‐cell ribotype CN and temperature. Our empirical data and modeling on single‐cell ribotype scaling are in agreement with both the metabolic theory of ecology and the growth rate hypothesis, providing a quantitative framework for linking cellular rDNA and rRNA CNs with body size, growth (activity), and biomass stoichiometry. This study also demonstrates that the expression rate of rRNA genes is constrained by cell size, and favors biomass rather than abundance‐based interpretation of quantitative ribotype data in population and community ecology of protists.

RIBOSOMES constitute the most important molecular machine of the cell and are responsible for protein synthesis and cell growth. The ribosomal RNA gene (rDNA) encodes ribosomal RNA (rRNA), which makes up 50–60% of ribosomes. The small‐subunit ribosomal RNA genes (e.g., 16S and 18S rDNA) are the most popular molecular markers in phylogenetics, and have been used extensively in studies of microbial diversity and ecology. Structurally, rDNA clusters comprise 1–15 tandem repeats in prokaryotes (Stoddard et al. 2015), and hundreds to tens of thousands duplicated arrays in plant and animal genomes (Prokopowich et al. 2003). Recent studies have shown that the number of rDNA copies in unicellular eukaryotes (protists) varies even more greatly, ranging from only a few in picoeukaryotes to hundreds of thousands in large‐sized protists, such as dinoflagellates, diatoms, and ciliates (Galluzzi et al. 2004; Godhe et al. 2008; Gong et al. 2013; Massana et al. 2008; Rodríguez‐Martínez et al. 2009; Torres‐Machorro et al. 2010; Zhu et al. 2005). The varying copy number (CN) of rDNA between microbial taxa can introduce systematic bias in assessing rDNA‐based diversity and community composition (Gong et al. 2013; Medinger et al. 2010; Thornhill et al. 2007; Weber and Pawlowski 2013). While the bias can be largely corrected by using the ribosomal RNA operon CN database for bacteria and archaea (Stoddard et al. 2015), such a database is not available for protists so far. Thus, properly interpreting rDNA‐based gene abundance remains a critical issue in molecular ecology of protists.

The rDNA CN varies widely within and between eukaryotic species (Rogers and Bendich 1987; Weider et al. 2005) rendering ecological inferences of rDNA CN‐based eukaryotic cell numbers difficult. The large rDNA CN variations (CNVs) observed in some plants across latitudinal, longitudinal, and elevational gradients have been attributed to environmental effects (e.g., temperature, fire‐related stress) (Bobola et al. 1992; Govindaraju and Cullis 1992; Strauss and Tsai 1988). Varying amounts of cellular rDNA contents have been found in different growth phases, or among isolates and strains of foraminifera, dinoflagellates and fungi (e.g., Galluzzi et al. 2010; Herrera et al. 2009; Parfrey et al. 2012). A linear correlation between rDNA CN and cell length or cell volume (CV) has also been demonstrated in some protists (Godhe et al. 2008; Zhu et al. 2005). However, the cause and relationship between environmental factors and protistan rDNA CNs, as well as, the linkage between rDNA CN and phenotypic traits (e.g., cell size and growth rate) at the organismic level, are not well understood. Furthermore, previous studies mainly used pooled cells, thus a fundamental understanding of rDNA CNVs at the single‐cell level is still lacking.

Relative to rDNA, its expressed product, rRNA or ribosome, has been commonly associated with the physiological status and growth rates of bacteria and animals. Nonetheless, only a few studies have investigated the relationship between cellular RNA content and growth rate of protists. While the total cellular RNA and rRNA contents varied in some microalgae (Berdelet et al. 1994; Dortch et al. 1984; Taylor et al. 2014), it did not differ significantly in others (Medlin and Kegel 2014). For instance, the RNA content of the dinoflagellate *Alexandrium* was not significantly correlated with the growth rate under changing conditions (Taylor et al. 2014). Hence, the use of rRNA or expression rates of the rRNA genes (rRNA:rDNA ratios) as a proxy for protistan activity is questionable, as already reviewed by Blazewicz et al. (2013). Utilizing rRNA‐related parameters to characterize protistan communities requires a better understanding of the quantitative relationships of these parameters with rDNA, body size, and growth rate under environmental changes.

In this study, we used the ciliated protozoa *Euplotes vannus* and *Strombidium sulcatum* to simultaneously investigate the CNVs of rDNA and rRNA/cDNA (reverse‐transcribed from rRNA) and how they associate to changes in body size and growth rate along a temperature gradient. Temperature was selected because it is the most important environmental factor influencing biological processes and reflecting seasonal cycles, geographic distribution, and climate change. Our empirical data and modeling linking ribotypes and phenotypic traits have important implications for interpreting ribotype data in studies focusing on diversity and ecology of protists.

## Materials and Methods

### Protistan cultivation and treatments

Two ciliate protists *E. vannus* and *S. sulcatum*, originally isolated from the coastal zone of Qingdao, were maintained in Petri dishes with filtered seawater (salinity 30 PSU) and several rice grains to enrich bacterial growth. These cultures have been maintained at 16 °C for over 10 yr in the ciliate collection of the Laboratory of Protozoology, Ocean University of China. In a pilot study, we established a series of batch cultures of these two species, and confirmed that they could grow between approximately 14 and 26 °C (data not shown). To set up growth experiments, individual cells were picked up with a micropipette and repeatedly washed to remove other protists and bacteria. The cleaned protists were inoculated into a Petri dish with 20 ml of culture medium consisting of a mixture of sterilized seawater and 0.1 ml heat‐killed (70 °C for 10 min) cells of *Escherichia coli*. Protistan cultivation was carried out in three cabinets under constant humidity but different temperatures at 16, 21, and 25 °C (thereafter referred to as the “warming” treatments T16, T21, and T25, respectively). During a period of 30 d, approximately 10 cells from each of these warming treatments were re‐inoculated into fresh medium every week to allow growth without resource limitation in the first few days. In order to test for possible footprint effect of the higher temperature on ribotypic traits, we set a “touchdown” treatment (T16*) for both species, whereby we transferred a few individuals from the T25 that had been maintained at 25 °C for 30 d and re‐cultured them at 16 °C for another month as described above. Triplicates were set up for each treatment.

### Determination of growth rate and cell size

Starting at the fourth inoculation, cell density was monitored every 24 h. In the middle of the exponential growth phase, about 100 μl of medium solution was taken out and fixed with Lugols solution (final concentration 2%). For each sample, at least 200 protistan cells were counted under a stereoscope. The natural log protistan abundance was plotted against time to generate a growth curve, and the data of the logarithmic growth phase were selected to calculate a linear regression formula, of which the slope was used to estimate the growth rate. At the exponential growth phase, cells were subsampled and fixed. Among these fixed cells, 24 individuals were randomly selected to measure cell width and length under a microscope (Olympus BX51, Tokyo, Japan). Cell volume was calculated assuming an elliptic cylinder shape for *E. vannus* with a body width to thickness ratio of 3:2, and an ellipsoid shape for *S. sulcatum*.

### Single‐cell nucleic acid extraction and cDNA synthesis

Sixteen cells (replicates) were set up in parallel for each treatment. For each replicate, a single cell was isolated, transferred into autoclaved seawater that had been filtered through a 0.2‐μm filter. The cell was repeatedly washed with a glass micropipette to minimize contamination. Finally, the cleaned cell was placed within a tiny drop of seawater (~0.5 μl) and transferred onto the sidewall of a nuclease‐free microcentrifuge tube (Axygen Scientific Inc, Silicon Valley, CA). The tube was inspected under a stereoscope to confirm presence and viability of the cell before adding reagents. Cell lysis solution containing 2 μl DEPC‐treated water (TaKaRa Biomedicals, Beijing, China), 0.3 μl 5% Nonidet P‐40 (RTM: Octyl phenoxylpolyethoxylethanol) (NP40) (Sangon, Shanghai, China), and 0.2 μl Recombinant RNase Inhibitor (TaKaRa Biomedicals) was added for simultaneous extraction of DNA and RNA from the single protistan cell. The tube was flicked and centrifuged briefly to let the cell settle at the bottom of the tube. After letting the tube stand for 5 min at room temperature, 1 μl of cell suspension was placed into a PCR tube for genomic DNA extraction using the RED Extract‐N‐Amp Tissue PCR Kit (Sigma, St. Louis, MO) according to the manufacturer's protocol, modified such that only 1/10 of the suggested volume for each solution was used (Gong et al. 2013).

Single‐cell cDNA synthesis was mainly according to Bengtsson et al. (2008) and Ståhlberg and Bengtsson (2010), with the following modifications: for reverse transcription, an extra 1 μl of cell suspension containing both DNA and RNA was taken to a separate tube and diluted to 9 μl. Two μl of random hexamers primer (10 mM; B0043, Sangon) and 1 μl dNTP (2.5 mM) were added immediately. Denaturation of the total RNA was executed within a water bath at 70 °C for 5 min. Reverse transcription reaction was performed with the SuperScript III Reverse Transcriptase Kit (Invitrogen, Shanghai, China). The total volume of the PCR reaction was 20 μl containing 4 μl of 5× first‐strand buffer, 0.3 μl of SuperScript III reverse transcriptase (200 U/μl), 1 μl of recombinant RNase inhibitor, 1 μl of 0.1 M DTT, and 3.2 μl DEPC‐treated water. The tube was immediately subjected to heating on a thermal cycler (Biometra, Göttingen, Germany) at 25 °C for 5 min, 50 °C for 50 min, and 85 °C for 50 min, and then transferred to an ice‐bath for 2 min. The DNA and cDNA was stored at −80 °C for subsequent analyses.

### Quantitative real‐time PCR

In the course of our study, we found out that the total rRNA in the single‐cell solution might become partially degraded, if the rDNA was completely digested by using a DNAase. To minimize RNA degradation, rDNA was not removed during cDNA synthesis. For the quantitative real‐time PCR (qPCR) assay, the specific primers EvQ‐F/R (5′‐CACTTCTACGGAAGGCAGCA‐3′/5′‐TGCCCTCCAATTGTTCCTCG‐3′) and SsQ‐F/R (5′‐CATGCTGGACAGCCTGACTT‐3′/5′‐CGTTTCTCAGGCTCCCTCTC‐3′) were newly designed to amplify fragments of 144 bp for *E. vannus* and 235 bp for *S. sulcatum*. For *Euplotes*, the plasmid standard curves showed a significant linear relationship between the threshold cycle (Ct) values and the rDNA copy number: Ct = −3.587 × log_10_ (rDNA copies/μl) + 38.64, with an efficiency of 90.0% and *R* ^2^ of 0.9989. For *Strombidium*, the linear relationship obtained with the standard was Ct = −3.426 × log_10_ (rDNA copies/μl) + 38.05, with an amplification efficiency of 95.8% and *R* ^2^ of 0.9991 (Fig. S1). Based on these standard curves and the Ct values obtained from each single cell, the rDNA copies per cell were estimated for each isolate at different temperatures. The rRNA/cDNA CNs were calculated by subtracting the rDNA CNs from the sum of rDNA and cDNA CNs. Solutions and qPCR protocol were as previously described (Gong et al. 2013).

### Statistical analysis

Student's *t‐*test (two‐tailed) was performed to test the null hypotheses that growth rates, cell volumes, and CNs differed between the various temperature treatments. Pearson's correlations and regression analysis were performed to explore the relationships among cell volume, growth rate, rDNA and rRNA CNs, and rRNA:rDNA ratio. All these analyses were executed using SPSS v.13.0 (SPSS, Chicago, IL).

## Results

### Changes in growth rate and cell size at different temperatures

**Figure 1:**
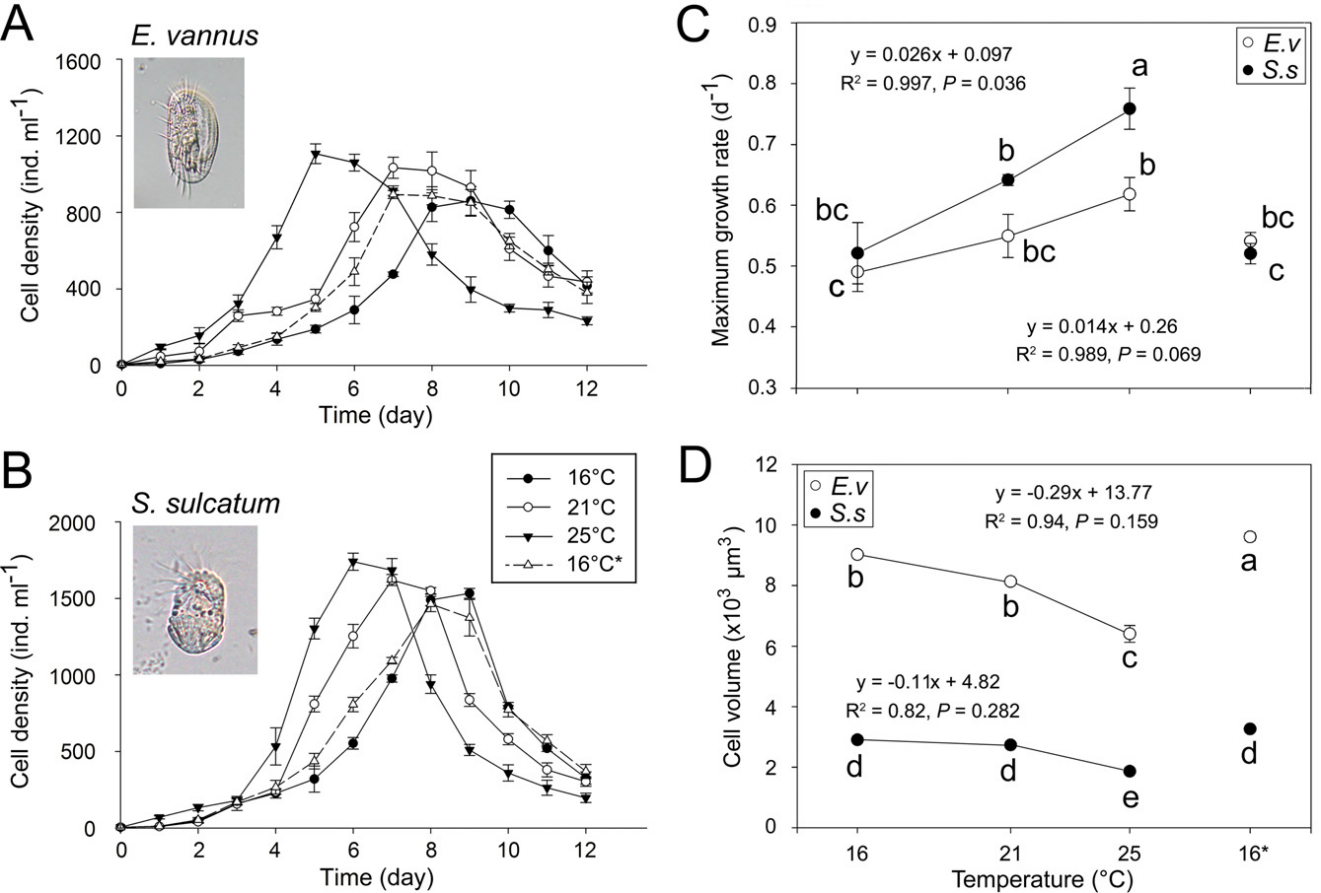
Growth curves (**A, B**), maximum growth rates (**C**), and cell volumes (**D**) of *Euplotes vannus* (A) and *Strombidium sulcatum* (B) in different temperature treatments. Two treatments sharing no lowercase letters indicate significant differences (*P *<* *0.05). The “16*” denotes the touchdown treatment that was re‐established by transferring a few individuals from the treatment maintained at 25 °C to a new culture incubated at 16 °C.

Growth rates and cell sizes were determined for the warming and touchdown treatments of both *E. vannus* and *S. sulcatum* (Fig. 1). At T16, T21, and T25, the average growth rates were 0.49 d^−1^, 0.55 d^−1^, and 0.62 d^−1^ for *E. vannus* and 0.52 d^−1^, 0.64 d^−1^, and 0.78 d^−1^ for *S. sulcatum*, showing that the growth rate of both species increased significantly with increasing temperature (*P *<* *0.05; Fig. 1C). Growth rates of both species at the touchdown treatment (T16*), were not significantly different from those at T16 (*P > *0.05). *Strombidium sulcatum* grew significantly faster than *E. vannus* at T21 and T25 treatments, but not at T16 and T16*, suggesting that the response of growth to warming is species dependent. Between 16 and 25 °C, the growth rates of *E. vannus* and *S. sulcatum* increased by 0.014 ± 0.0023 d^−1^ °C^−1^ (mean ± SE) and 0.026 ± 0.0072 d^−1^ °C^−1^, respectively, suggesting that the growth of the latter is more sensitive to warming.

The cell size generally shrank with increasing temperature (Fig. 1D). For *E. vannus*, the mean CV changed by −2.0% (95% confidence intervals, CIs ± 0.59%) per °C from 16 to 21 °C, −4.2% (CIs ± 1.58%) per °C from 21 to 25 °C, and −3.2% (CIs ± 0.66%) per °C from 16 to 25 °C. The CV of *S. sulcatum* decreased sharply at higher temperatures, with rates of −1.2% (CIs ± 0.59%) per °C from 16 to 21 °C, −6.4% (CIs ± 0.22%) per °C from 21 to 25 °C, and −4.0% (CIs ± 0.12%) per °C from 16 to 25 °C. While the cell size of *S. sulcatum* at T16* was not significantly different from that at T16, that of *E. vannus* was larger by 12.8% (*P *<* *0.05).

### Single‐cell rDNA and rRNA CNVs

**Figure 2:**
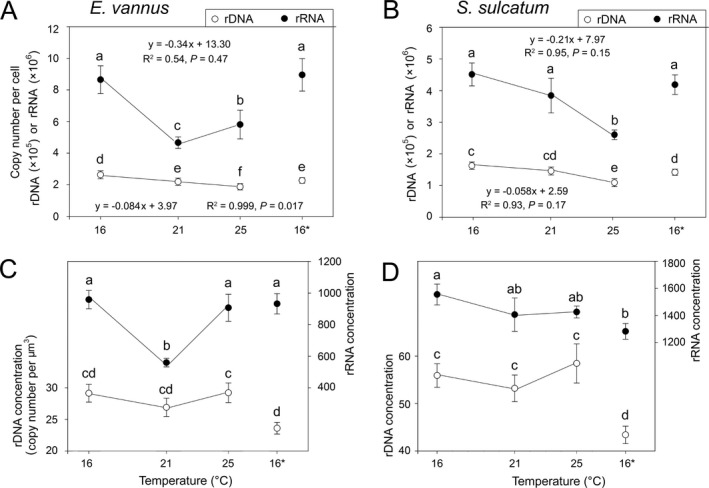
Single‐cell 18S rDNA and rRNA/cDNA copy number (**A, B**) and concentration (**C, D**) variations in *Euplotes vannus* and *Strombidium sulcatum* among temperature treatments. Two treatments sharing no lowercase letters indicate significant differences (*P *<* *0.05). The “16*” denotes the touchdown treatment that was re‐established by transferring a few individuals from the treatment maintained at 25 °C to a new culture incubated at 16 °C.

The qPCR results showed that the rDNA CN per cell (CNPC) was higher in *Euplotes* [(2.6 ± 0.25) × 10^5^] than in *Strombidium* [(1.6 ± 0.12) × 10^5^] at the T16 treatment (Fig. 2A, B). With increasing temperature, the rDNA CNs of both species gradually decreased at T21 and T25, with slopes of about −8,420 copies/°C (−3.20%/°C) and −5,943 copies/°C (−3.66%/°C) for *Euplotes* and *Strombidium*, respectively. Compared with T16, the mean rDNA contents at T16* appeared lower by 13.7% and 12.7% in these two species (*P *<* *0.05).

The rRNA (cDNA) was markedly more abundant than rDNA in single cells of both species across all temperature treatments (Fig. 2A, B). At T16, *E. vannus* and *S. sulcatum* had on average 8.6 × 10^6^ and 4.5 × 10^6^ rRNA copies/cell, corresponding to 33 and 28 times as many as those of rDNA, respectively. Similar to rDNA, the rRNA transcript numbers also decreased at higher temperatures (T21 and T25). The slopes were much steeper, about −3.65%/°C in *E. vannus* and −4.70%/°C in *S. sulcatum*, indicating high and species‐dependent sensitivity of rRNA content to warming. At T16*, rRNA contents were significantly lower than those at T25 (*P *<* *0.05). Nevertheless, the rRNA CNs were not significantly different between the T16* and T16 treatments (*P *>* *0.05).

The cellular concentrations of rDNA and rRNA ([rDNA] and [rRNA]) of both species were relatively stable across warming treatments (Fig. 2C, D). Nevertheless, *Strombidium* had a much higher concentration of both rDNA (mean 56 vs. 29 copies/μm^3^) and rRNA (1,455 vs. 813 copies/μm^3^), when compared to *Euplotes*. Generally, at the T16* treatment, both [rDNA] and [rRNA] were significantly lower than at T16, except for the [rRNA] in *Euplotes*, which had a similar level with that at T16.

**Figure 3:**
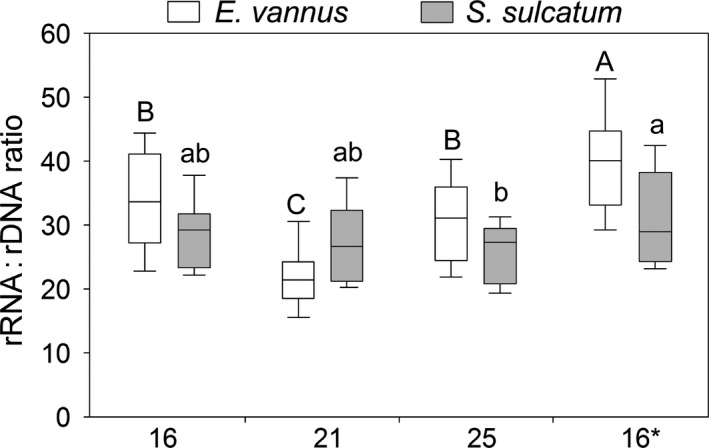
Comparisons of rRNA‐to‐rDNA copy number ratios among temperature treatments of the ciliates *Euplotes vannus* and *Strombidium sulcatum*. The “16*” denotes the touchdown treatment that was re‐established by transferring a few individuals from the treatment maintained at 25 °C to a new culture incubated at 16 °C. Two treatments sharing no lowercase or uppercase letters indicate significant differences (*P *<* *0.05).

Overall, the ratios of rRNA:rDNA in the warming treatments varied within narrow ranges, with generally higher levels in *Euplotes* (21.3–32.9) than in *Strombidium* (23.9–27.8). Among these treatments, the rRNA:rDNA ratios were not significantly different, except in the case of *E. vannus* at T21, whereby the ratio appeared much lower (*P *<* *0.05). The ratio at T16* (39.5) was much higher than that at T16 (32.9) for the *Euplotes* species, a pattern, however, that was not observed for *Strombidium* (Fig. 3).

### Correlations and modeling between genotypic and phenotypic traits

**Figure 4:**
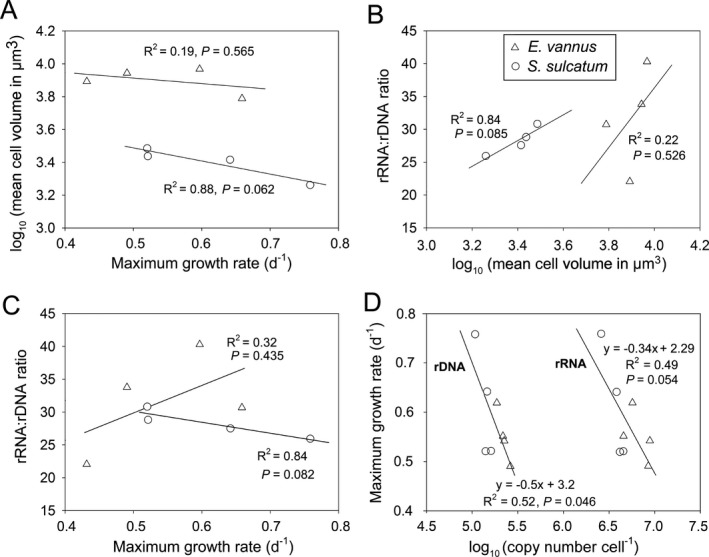
Pearson's correlations and regressions between phenotypic traits and ribotypic attributes of two ciliate species reared at different temperatures. (**A**) Growth rate and cell volume; (**B**) cell volume and rRNA: rDNA ratio; (**C**) growth rate and rRNA: rDNA ratio; (**D**) cellular rDNA and rRNA copy numbers and growth rate.

To explore possible quantitative links between genotypic and phenotypic traits, the data derived from the four treatments were subjected to correlation and/or linear regression analyses (Fig. 4). Not surprisingly, the mean CV decreased with increasing growth rate, although this relationship was only marginally supported for *Strombidium* (Fig. 4A). Similarly, a positive correlation between the rRNA:rDNA CN ratio and the mean CV was marginally supported for *Strombidium* (*R* ^2^ = 0.84, *P *<* *0.1), but not for *Euplotes* (*R* ^2^ = 0.22, *P *>* *0.5) (Fig. 4B). The correlation between rRNA:rDNA ratio and growth rate was positive and weak for *Euplotes* (*R* ^2^ = 0.32), but negative for *Strombidium* (*R* ^2^ = 0.84) (Fig. 4C). Nevertheless, the per‐cell CNs of both ribotypes were negatively correlated with growth rate for both species, although the coefficient of determination was moderate in both cases (*R* ^2^ = 0.52 and 0.49; Fig. 4D).

**Figure 5:**
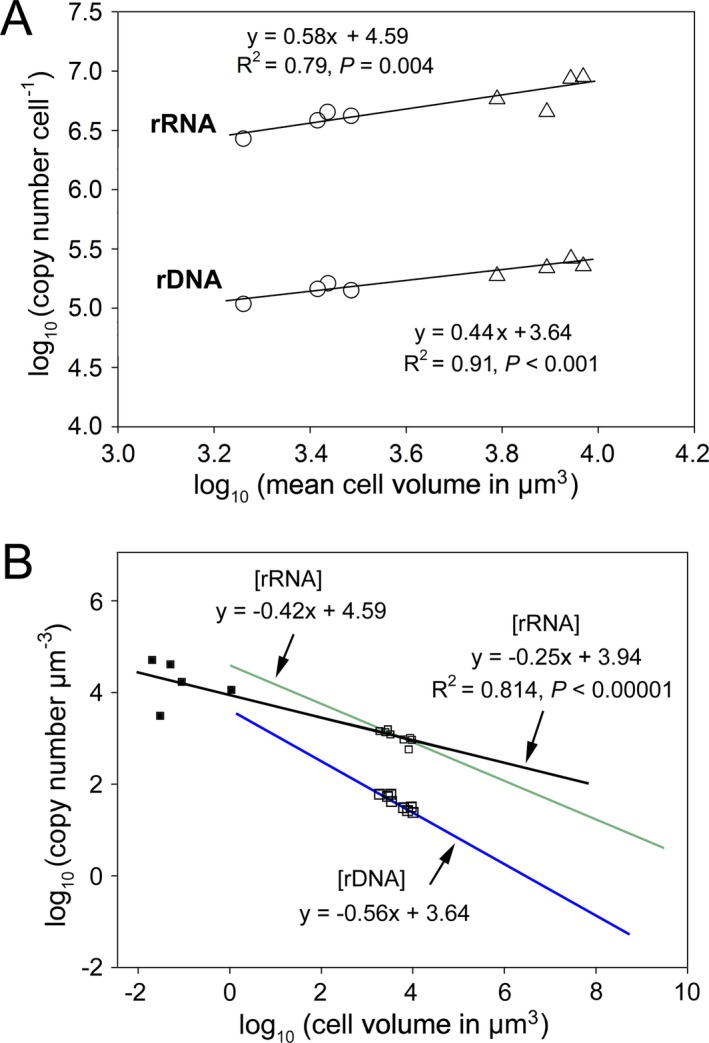
(**A**) Regression analysis of log‐transformed per‐cell copy numbers of rDNA and rRNA and log‐transformed cell volumes across two ciliate species reared at different temperatures. (**B**) Linear relationships between cell volume and rDNA and rRNA concentrations in a log–log plot. The relationship based on ciliate rDNA and rRNA data obtained in this study follows *y* = −0.56*x* + 3.64 (blue line) and *y* = −0.42*x* + 4.59 (green line), respectively. When the ribosomal number per cell and cell volumes of the five prokaryotic species, *Rickettsia prowazekii*,* Escherichia coli* (from Pang and Winkler 1994); *Sphingomonas* sp. strain RB2256, (from Fegatella et al. 1998); *Spiroplasma melliferum* (from Ortiz et al. 2006); and a tiny archaeon named ARMAN (from Comolli et al. 2009), were incorporated for regression analysis, the function turns to *y* = −0.25*x* + 3.94 (black line).

The most consistent and significant correlations were found between ribotype CNs and cell size (Fig. 5A). The log‐transformed mean CV was linearly correlated with log rDNA (*R* ^2^ = 0.91, *P *<* *0.001) and rRNA CNPC (*R* ^2^ = 0.77, *P *=* *0.004; Fig. 5A). In these relationships, the per‐cell CN of rDNA and rRNA increases with increasing CV (in volume units of μm^3^), according to power functions depicted as follows:

(1) $$\text{rDNA CNPC}=4365\times {\text{CV}}^{0.44}$$

(2) $$\text{rRNA CNPC}=38905\times {\text{CV}}^{0.58}$$

So that [rDNA] and [rRNA] follow power law functions (Fig. 5B):

(3) $$\text{[rDNA]}=4365\times {\text{CV}}^{-0.56}$$

(4) $$\text{[rRNA]}=38905\times {\text{CV}}^{-0.42}$$

When data of ribosome (rRNA) content from five bacterial and archaeal species were incorporated, the equation for rRNA CN per μm^3^ changed into the equation below (5) (Fig. 5B):

(5) $$\text{[rRNA]}=8710\times {\text{CV}}^{-0.25}$$

with *R* ^2^ = 0.814, *P *=* *2.5E‐05, *n* = 13.

**Figure 6:**
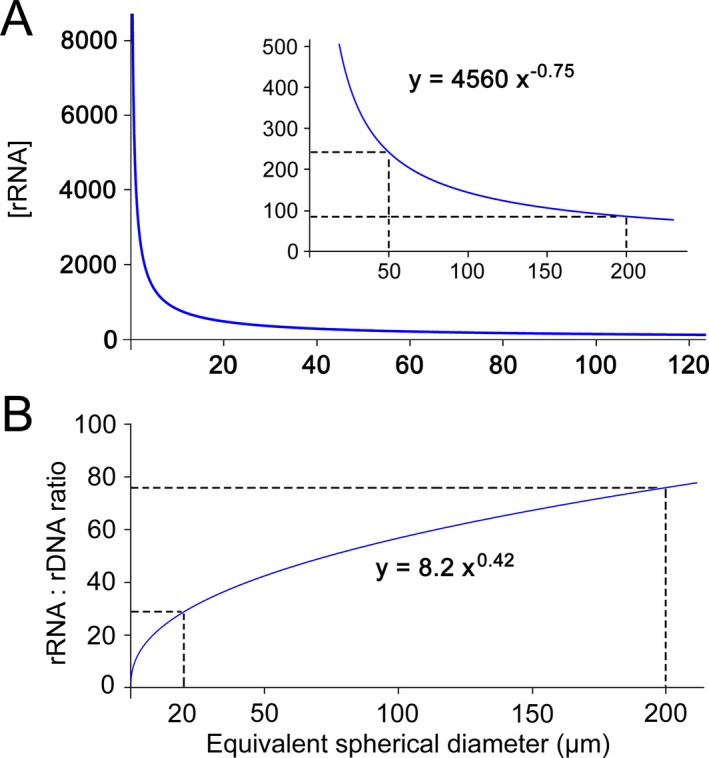
Modeled size scaling relationships of cellular rRNA concentration (copies per μm^3^) and rRNA: rDNA ratio. (**A**) Variations in [rRNA] along cell size (equivalent spherical diameter) spectrum predicted based on five bacteria and ciliate data. (**B**) An extrapolation for the relationship between rRNA: rDNA ratio and protistan equivalent spherical diameter using the ciliate data obtained in this study.

The CN ratio of rRNA to rDNA in a single cell is calculated by:

(6) $$\text{rRNA:rDNA}=9\times {\text{CV}}^{0.14}$$

(7) $$\text{Or rRNA:rDNA}=8.2\times {\text{ESD}}^{0.42}$$

where ESD is the equivalent spherical diameter of a cell (Fig. 6B).

Montagnes et al. (1988) developed a multiple‐regression model to predict ciliate growth rate by incorporating both temperature and cell size:

(8) lnr=at−blnCV−c

where *r* is the maximum growth rate (d^−1^); *t* is temperature (°C) and *a*,* b*, and *c* are constants. Accordingly, the linear scaling between ln (ribotype CNPC) and ln(CV) as shown in equations ((1) and (2)), *r* can also be modeled by the following equation:

(9) lnr=at−bln(ribotype CNPC)−c

Using the data obtained in the present study, *a*,* b*, and *c* were estimated as follows:

(10) lnr=0.024t−0.208ln(rDNA CNPC)+1.484

(11) lnr=0.024t−0.116ln(rRNA CNPC)+0.762

The coefficients of determination *R* ^2^ (adjusted *R* ^2^) were 0.882 (0.834) for equation (10), and 0.815 (0.741) for equation (11).

## Discussion

### Temperature selects body size and rDNA copy number variants of protists

Due to the single‐cell nature of protists, plasticity in morphology, physiology, and life cycles at an organismal level also represent strictly cellular responses. Phenotypic changes that are temperature dependent have been well documented in protists (e.g., Atkinson et al. 2003; Daufresnea et al. 2009; Fenchel 1968; Finlay 1977; Forster et al. 2013; Montagnes and Franklin 2001; Montagnes et al. 2003). Our observation of the inverse relationship between body size of the two heterotrophic protist species and temperature is consistent with the *temperature–size rule* (TSR), revealed in a meta‐analysis study of protistan taxa across habitats (marine, brackish, or fresh water) and modes of nutrition (heterotrophs or autotrophs) (Atkinson et al. 2003). The slopes of size‐decrease (−3.2 and −4.0%/°C) recorded in our study are comparable to these previously reported by Atkinson et al. (2003), who also reported that cell size decreases linearly with increasing temperature at on average of 2.5% (CIs ± 0.8%)/°C of the volume observed at 15 °C. In addition, we observed that both species used in this study could revert to their original cell sizes, when the culturing temperature decreased from 25 °C to 16 °C, further highlighting the high phenotypic plasticity of ciliate cells at changing temperatures.

Previously, we employed single‐cell qPCR to accurately determine the per‐cell rDNA CNs in a range of ciliates. We demonstrated that the rDNA content per cell is generally much higher in ciliates than in other eukaryotic taxa, and that interspecies differences in rDNA content could be substantial (Gong et al. 2013). The results of the present work further support those findings. Herein, we take a step forward by providing compelling evidence that environmental temperature plays an important role in selecting for rDNA CN variants within a protistan species. We show a negative relationship between cellular rDNA CN at mid‐log growing phase of ciliate protists and temperature under food‐replete conditions (Fig. 2A, B). This experimental result is consistent with previous observations on plant species (e.g., Douglas fir and spruces): plants growing at higher latitudes and lower temperatures had higher rDNA CNs (Bobola et al. 1992; Strauss and Tsai 1988). Collectively, these findings suggest that: (1) the per‐cell rDNA content could be a genetic/genomic trait reflecting species‐, strain‐, or ecotype‐level adaptations of eukaryotes to local temperature or climate, and (2) warming tends to select protistan cells with fewer rDNA operons in their genomes. Nevertheless, Galluzzi et al. (2010) cultured different strains of *Alexandrium catenella* that were originally isolated from separate locations along the Mediterranean Sea and measured rDNA CN in exponentially growing cells in pure cultures at an identical temperature (20 °C). They found significantly different rDNA CNs among the *A. catenella* strains and these differences were not related to geographical origin. This suggests that a single protistan species may have many ecotypes distinguished by variable rDNA CN that co‐exist in a given natural environment to maintain high micro‐diversity at certain spatial scales. It is likely that the protistan ecotypes with lower rDNA CN are dominant members of the natural community at higher temperatures, but this has yet to be tested.

### Both genomic rDNA and cellular rRNA copy numbers exponentially scale with cell size, but with different exponents

The enormous variation in the rDNA CN within and between eukaryotic genomes has puzzled researchers for a long time. This is, in part, because the total number of rDNA copies is well beyond the number necessary to maintain a sufficient supply of rRNA transcripts, and some copies remain transcriptionally inactive even at maximum growth stages (Reeder 1999). Hence, the extensive interspecies variability in rDNA copy number cannot be completely explained by differences in transcriptional requirements. Recent studies have found that rDNA CN is associated with genome size (Prokopowich et al. 2003), genome stability (Ide et al. 2010), genome‐wide gene expression (Paredes et al. 2011), and mitochondrial abundance (Gibbons et al. 2014). Collectively, these studies hint at a link between the evolution of rDNA CN and cell size, but relevant data for this association has been sparse.

The single‐cell qPCR method used in this study allowed us to determine the CN of rDNA and rRNA molecules with a high resolution, which facilitates linking of ribotype CNs to phenotypic traits at the individual level. Our study is the first to demonstrate strong cell size scaling of rDNA copies in actively growing eukaryotic organisms along a fully manipulated temperature gradient. This scaling relationship holds within and across two ciliate species (Fig. 5A). These findings, along with previous observations regarding correlation between rDNA CN and cell length or CV in several protist taxa (Godhe et al. 2008; Zhu et al. 2005), suggest that cell size poses a strong constraint on the rDNA CN in protists. This can be easily understood because rDNA CN is positively correlated with genome size (Prokopowich et al. 2003), whereas genome size is positively correlated with cell volume across eukaryotic species (e.g., Cavalier‐Smith 1982; Connolly et al. 2008). Functionally, very low rDNA CNs in chicken stains could cause embryonic lethality (Delany et al. 1994), whereas many extra (untranscribed) rDNA copies are required to facilitate condensin association and sister‐chromatid cohesion, thus allowing efficient replication‐coupled recombinational repair in the yeast *Saccharomyces cerevisiae* (Ide et al. 2010). This partly explains the increase in rDNA CN when the ciliate cells became larger after transferring them from T25 to T16* treatments. The mechanism of enrichment of rDNA copies could be via extrachromosomal amplification in many single‐celled eukaryotes (e.g., ciliates, excavates, amoebae, and fungi; see Torres‐Machorro et al. 2010), or via whole genome duplication and tandem arrangement in other eukaryotes (Rogers and Bendich 1987). Still, the question remains as to why a high content of rDNA was not maintained when cells became smaller. A potential explanation is that cells select for balanced growth, metabolic, and spatial economy by optimizing karyoplasmic volume ratios (Cavalier‐Smith 2005). It is thus not difficult to imagine that the nucleoli, being the largest nuclear compartments in the nucleus and the site of rDNA sequences, ribosome synthesis, and assembly, may also be limited by the nuclear volume and may eventually become constrained by the overall cell volume.

The power law function for the rDNA CN and ciliate cell size relationship we obtained in this study may not be applicable for prokaryotes and some small‐sized eukaryotes. According to equation (1) [genomic rDNA CN = 4365 × CV^0.44^], bacteria with an ESD ranging from 0.2 to 2 μm could have 390–8,200 rDNA copies in their genome, a number that is a hundred times higher than the ones already determined for many prokaryotes (Stoddard et al. 2015). This discrepancy between bacterial and eukaryotic domains could stem from their differences in cellular and genomic size variations (limited and stable vs. large and dynamic) (Parfrey et al. 2008), as well as, spatial packing of rDNA molecules (not nucleus‐constrained vs. enclosed in nucleoli). Some picoeukaryotes were reported to have about 4–30 rDNA copies (Massana et al. 2008; Rodríguez‐Martínez et al. 2009; Zhu et al. 2005), a number that is much lower than our ciliate‐based predictions. Therefore, the cell volumes and the corresponding rDNA CNs of more taxa have to be characterized in order to refine their quantitative relationship in protists.

### Cell size poses constraints on rRNA concentration, growth rate, biomass stoichiometry, and beyond

To our knowledge, this study is the first to quantify rRNA CNs in single cells of protists, although cellular total RNA or rRNA contents (mass concentration) have been previously quantified based on pooled cells for a number of protistan species (e.g., Berdelet et al. 1994; Dortch et al. 1984; Medlin and Kegel 2014; Taylor et al. 2014). Our results show that cellular rRNA CN in these growing ciliates scales with cell volume to the 0.58th power, which is higher than the 0.44th power for rDNA cell size scaling (Fig. 5A). This rRNA CN‐cell volume scaling is comparable to the one previously established for ribosome amount and body mass in animals and yeasts (Gillooly et al. 2005). Interestingly, when our and existing data from five heterotrophic prokaryotes were merged for the regression analysis, the newly obtained exponents for cellular rRNA amount and concentration changed from 0.58 to 0.75 and from −0.42 to −0.25, respectively (Fig. 5B), both of which are identical to the ones previously estimated by Gillooly et al. (2005), and are consistent with the metabolic theory of ecology (MTE, Brown et al. 2004). Thus, our empirical study provides evidence that the scaling relationship between rRNA/ribosome content and cell size is also valid in heterotrophic prokaryotes and protists, the major players in the microbial loop.

Since RNA is rich in phosphorus (P) and rRNA usually comprises over 80% of the total cellular RNA, more abundant rRNA transcripts (ribosomes) give rise to rapid protein synthesis for cellular growth. Hence, a higher rRNA concentration in an organism has been connected to a higher growth rate and lower biomass carbon (C) to P and nitrogen (N) to P ratios, known as the growth rate hypothesis (GRH) (Elser et al. 1996, 2000). The GRH was formulated for organisms growing at a given temperature, and has been tested mainly regarding the growth rate and P content (or P:C) relationships in multicellular crustacean zooplankton, insects, and bacteria. However, there are not many studies that examine the growth rate—[rRNA] relationship in protists (Hessen et al. 2013; Makino et al. 2003; Simonds et al. 2010). Our study of two heterotrophic ciliated protists at different temperatures shows that smaller cells with higher growth rates also have higher [rRNA], hence is supportive for the GRH. This indicates that the GRH of biological stoichiometry is largely consistent with MTE in both heterotrophic prokaryotes and protists, which provides an important and useful framework that integrates both theories in the context of molecular microbial ecology and global climate change.

Biomass stoichiometry, trophic interactions of heterotrophic bacteria and protozoa and nutrient cycling can be better understood when referring to the prediction that cell size constrains cellular [rRNA]. If we regard cellular C content as a simple function of cell size, then variations in cellular P:C ratio along the cell size spectrum should be similar to the curve of [rRNA] (Fig. 6A). Consequently, due to the small cell volume and size range of bacteria, their P:C ratio will be remarkably higher and more variable than that of protists. This size‐based prediction is supported by widespread empirical evidence for high levels and flexibility of elemental stoichiometry in heterotrophic bacteria (Chrzanowski and Grover 2008; Cotner et al. 2006; Godwin and Cotner 2015; Makino et al. 2003), and the cyanobacterial *Synechococcus* (Garcia et al. 2016) as well as higher P:C ratios in soil (1:60) and marine (1:77) bacterial biomass relative to the Redfield ratio of phytoplankton (1:106) (Cleveland and Liptzin 2007; Zimmerman et al. 2014). Cell size‐related P:C variations were also observed in a mixotrophic bacterivore (Simonds et al. 2010). Compared with bacteria, nano‐ and microplanktonic protozoa (> 2 μm in ESD) will be far less variable in P:C ratio within and among species (Fig. 6A), suggesting that stoichiometric homeostasis is cell size‐dependent and generally contrary between these two domains of microbes. As small cells are more nutrient‐rich than larger ones, the latter can get enough nutrients by consuming the former. Thus, it is easy to understand why (heterotrophic) grazers excrete excessive nutrients (i.e., nutrient regeneration) and are largely capable of maintaining stoichiometric stability. Under the global warming scenario and at warmer locations, the mean microbial cell size is expected to become smaller, resulting in high demand for P and more occurrences of P limitations. This could also explain the observed geographic pattern, whereby the P:C ratio of marine algae decreases with latitude and increases with the average sea‐surface temperature (inversely related with cell size) (Yvon‐Durocher et al. 2015).

### Both intra‐ and interspecific differences in cellular rRNA:rDNA are cell size‐dependent

There has been an increasing number of studies on using both rDNA and rRNA as markers to infer active or inactive components of protistan communities (e.g., Jones and Lennon 2010; Logares et al. 2014). Consistent with Blazewicz et al. (2013), our study demonstrates that rRNA:rDNA ratio is not a good indicator for protistan activity, since maximum growth rates were not correlated with the cellular rRNA:rDNA ratios in these two ciliates (Fig. 4C). We also found that the rRNA:rDNA ratio in actively growing ciliate cells scaled with cell volume to the power of a low value (0.14), hence the larger the cell size, the higher the rRNA:rDNA ratio (Fig. 6B). Consequently, at actively growing phases, larger species will generally account for a higher proportion in the rRNA library (rRNA%) than in the rDNA pool (rDNA%) of a community. This inference is supported by some observations that large‐celled groups (e.g., radiolarians, diatoms, and ciliates) frequently exhibit high rRNA%: rDNA% ratio in protistan communities (Hu et al. 2016; Lepère et al. 2016; Stecher et al. 2016). It should be noted, however, that the large‐celled radiolarians and marine alveolates (MALVs) were over‐represented in rDNA libraries but under‐represented in rRNA datasets, while smaller organisms like the marine stramenopiles (MAST), the choanoflagellates or the ancyromonads presented the opposite trend (Hu et al. 2016; Massana et al. 2008, 2015; Not et al. 2009; Stoeck et al. 2007). These discrepancies with our active growth and cell size‐based inference may be truly attributed to the dormancy of the large cells and the active growth of the small species. Thus, our finding challenges the notion that populations with larger cell sizes and higher rRNA%: rDNA% ratios are more active, or that the smaller ones with lower rRNA%: rDNA% ratios are inactive or dormant. Instead, it is more likely that large protists grow more slowly than tiny ones when resources are replete. We argue that the effect of body size must be taken into account when using rRNA data to infer the relative activity of two populations within a protist community or activity variations of a taxonomic group in different protist communities across time and space.

### Concluding remarks

The advent of sequencing technologies has transformed the capabilities of microbial ecology from measurements of cell abundance and biomass to molecular‐based science, in which rDNA and rRNA sequences can be quantitatively analyzed to determine protistan community composition and structure. It is becoming increasingly clear that the distribution of genes and correlations with physicochemical factors across time and space cannot be well understood from an evolutionary adaptive perspective, without linking ribotype traits with cell phenotypes (Falkowski and Oliver 2007). Based on empirical data and modeling, our study linked cellular ribotype copy number polymorphisms to phenology (cell size, fitness/growth rate). We demonstrated the combined effects of body size and temperature, which impose constraints on copy numbers and concentrations of both rDNA and rRNA, as well as their ratios in a protist cell. These single‐cell‐based findings may have important implications when investigating population and community ecology of protists using rDNA and rRNA as molecular markers:

1. the ribotype quantity of a population is more closely related to that population's biomass rather than cell abundance, especially when there is large variation in cell size within this population;
2. the relative proportion of a population, or between two populations, within a protist community reflects their proportion in biomass rather than cell abundance;
3. the diversity indices involving the relative abundance of ribotypes of species (e.g., Shannon and Simpson diversities) may also reflect biomass‐based diversity of protist communities; and
4. the effect of body size has to be considered when interpreting rRNA:rDNA data as a function of protistan activities.

This study also illustrates that the MTE and the GRH of biological stoichiometry theory can be integrated when examining heterotrophic protists. Nevertheless, it should be noted that the GRH might not apply in a robust manner in photoautotrophic microbes (Flynn et al. 2010), for which variations in cellular ribotypic numbers within and among taxa need to be characterized. Protistan ribotype and phenotype plasticity at different growth stages and under different nutrient/resource conditions should be further investigated. Having such a refined quantitative framework, may enable us to use ribotype‐based molecular tools to better understand and predict variations in phenotypic diversity, community, and microbial food web organization, as well as, ecological functions of cultivated and uncultivated microbes in diverse ecosystems under global climate changes.

## Conflict of Interest

The authors declare no conflict of interest.

## Supporting information


**Figure S1.** Standard curves of qPCR showing the linear relationships between plasmid concentrations of rDNA of two ciliate species, *Euplotes vannus* (**a**) and *Strombidium sulcatum* (**b**), and the numbers of threshold cycles (Ct).
