# Supplementary material for: Single Cell Analysis Linking Ribosomal (r)DNA and rRNA Copy Numbers to Cell Size and Growth Rate Provides Insights into Molecular Protistan Ecology
Source: J Eukaryot Microbiol. 2017 Jun 9;64(6):885–96. doi: 10.1111/jeu.12425 (PMC5697653; doi:10.1111/jeu.12425)

SUPPORTING INFORMATION

Single Cell Analysis Linking Ribosomal (r)DNA and rRNA Copy Numbers to Cell Size and Growth Rate Provides Insights into Molecular Protistan Ecology by Rao Fu & Jun Gong

Figure S1. Standard curves of qPCR showing the linear relationships between plasmid concentrations of rDNA of two ciliate species, *Euplotes vannus* (**a**) and *Strombidium sulcatum* (**b**), and the numbers of threshold cycles (Ct).

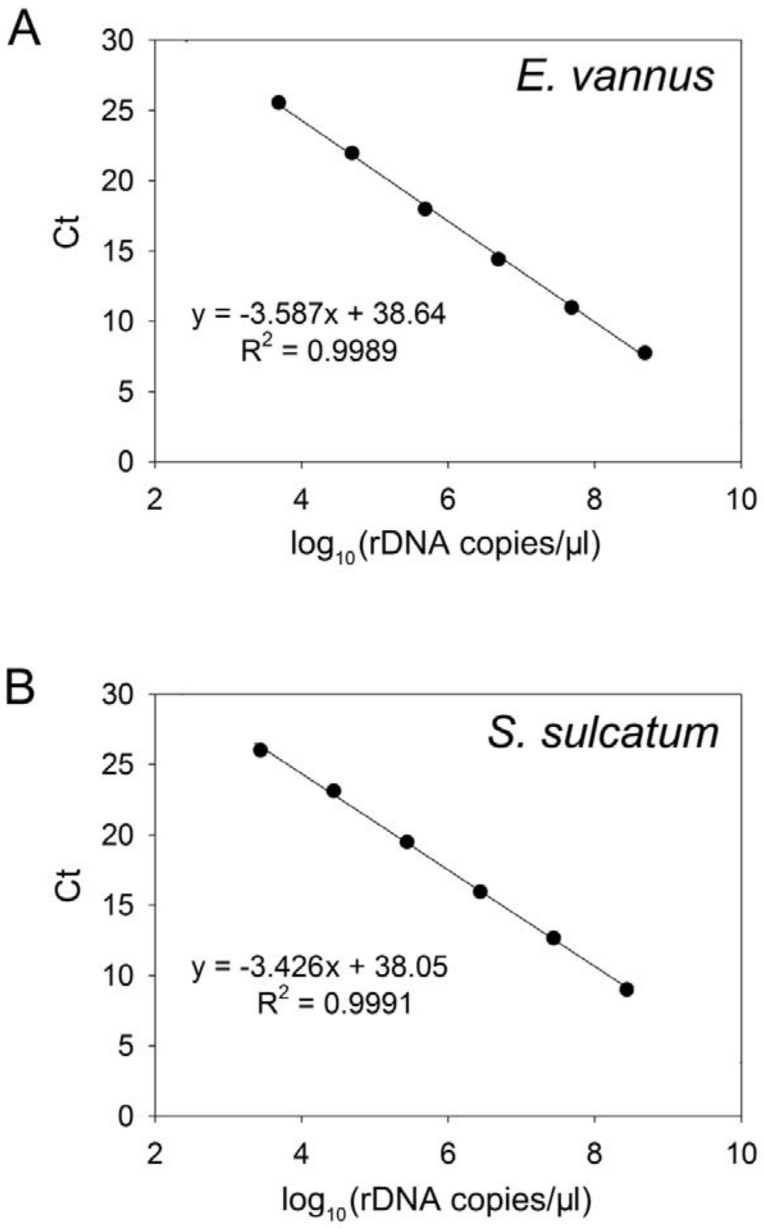

Supplement: Supplementary file 1 — Figure S1. Standard curves of qPCR showing the linear relationships between plasmid concentrations of rDNA of two ciliate species, Euplotes vannus (a) and Strombidium sulcatum (b), and the numbers of threshold cycles (Ct). [file JEU-64-885-s001.pdf]
